# Quantitative Muscle MRI as Outcome Measure in Patients With Becker Muscular Dystrophy—A 1-Year Follow-Up Study

**DOI:** 10.3389/fneur.2020.613489

**Published:** 2021-01-05

**Authors:** Aisha M. Sheikh, Karen Rudolf, Nanna Witting, John Vissing

**Affiliations:** Department of Neurology, Copenhagen Neuromuscular Center, Rigshospitalet, University of Copenhagen, Copenhagen, Denmark

**Keywords:** Becker muscular dystrophy, quantitative muscle MRI, muscle strength, fat fraction, outcome measure

## Abstract

**Introduction:** With the advent of emerging molecular therapies for muscular dystrophies, the need for knowledge about natural history course of such diseases is of utmost importance in the preparation for future trials. However, for Becker muscular dystrophy such knowledge is scarce.

**Objective:** In this 1-year follow-up study, we examined disease progression in Becker muscular dystrophy by monitoring changes in MRI-assessed muscle fat fraction (FF) in axial and lower limb muscles and quantitative muscle strength of axial muscles.

**Methods and Materials:** Sixteen patients with Becker muscular dystrophy were investigated by (1) muscle strength of the trunk using a Biodex dynamometer and (2) Dixon muscle MRI of paraspinal and lower limb muscles. Quantitative MRI data was analyzed in two parts: The first part consisted of all participants (*N* = 16). The second analysis assessed two separate groups comprising lesser affected participants (*N* = 5) and more severely affected patients (*n* = 11).

**Results:** Trunk extension and flexion strength remained stable from baseline to follow-up. MRI did not show any significant increase in muscle FF % from baseline to follow-up in all patients, except for multifidus at the spinal level T12 (*p* = 0.01). However, when we analyzed the two subgroups, according to disease severity, FF% increased in the lesser severely affected group at L4/L5 erector spinae (*p* = 0.047), sartorius (*p* = 0.028), gracilis (*p* = 0.009), tibialis anterior (*p* = 0.047), peroneals (*p* = 0.028), and gastrocnemius medialis (*p* = 0.009), while the severely affected group only increased significantly at T12 multifidus (*p* = 0.028) and T12 erector spinae (*p* = 0.011). No difference in muscle strength was observed in the two subgroups.

**Conclusion:** Our results add to the existing knowledge about the natural rate of disease progression in BMD. As quantitative MRI was able to identify changes where strength assessment was not, MRI could be a strong biomarker for change in BMD. However, our findings show that it is important to stratify patients with BMD according to phenotype for future clinical trials.

## Introduction

With the advent of emerging molecular therapies for muscular dystrophies, there is a need to know how the natural history of these diseases is, but for Becker muscular dystrophy such knowledge is scarce, especially with regard to axial muscles.

Previous imaging studies examining the lower limb muscles, using visual rating scales in studies of patients with BMD, have shown distinctive patterns of muscle involvement of the hamstrings, quadriceps, and gastrocnemius (1, 2). To quantify pathological changes and to monitor disease progression in myopathies, quantitative MRI (qMRI) technique is superior to visual rating scales to show change (3, 4). Quantitative strength assessment of the lower limb muscles and the muscles of the trunk using Biodex has been established to be a reliable method in previous studies (5, 6). With this 1-year follow-up study, we wish to gain knowledge on how axial muscle involvement progresses over time in BMD using quantitative muscle MRI.

## Methods and Materials

### Study Design and Participants

This prospective longitudinal study was conducted from April 2018 to June 2020 at Copenhagen Neuromuscular Center, Rigshospitalet, Copenhagen, Denmark, in accordance with the declaration of Helsinki and was approved by the Danish National Committee on Health Research Ethics (approval number: H-16030358).

We recruited 16 participants with genetically verified Becker muscular dystrophy from our Neuromuscular Center (Table 1). All 16 participants had previously participated in a cross-sectional study. Thirteen participants were able to ambulate without walking aid, while three were dependent on wheelchair for most functions. All participants were evaluated at baseline and at follow-up for trunk muscle strength and qMRI of the paraspinal and lower limb muscles. Muscle strength measure and qMRI were completed on the same day.

**Table 1:** Demographics of the 16 participating Becker muscular dystrophy patients.

**Subject ID**	**Age at** **follow-up**	**Age at** **symptom onset**	**Mutation**	**Disease duration** **in years**
BMD 1	46	28	c.676_678del; p.Lys226del	18
BMD 2	51	41	Del26	10
BMD 3	37	10	Del45-48	27
BMD 4	31	10	c.6912+1G>T	21
BMD 5	38	2	Del45-48	36
BMD 6	33	29	c.1602 G>A	4
BMD 7	28	24	Del45-47	4
BMD 8	34	5	Del45-48	29
BMD 9	19	1	c.5632C>T, p.(Gln1878^*^)	18
BMD 10	39	22	Del45-48	17
BMD 11	39	6	Del45-48	33
BMD 12	30	29	Del45-47	1
BMD 13	32	8	Del45-47	24
BMD 14	26	6	Del45-49	20
BMD 15	60	35	Del48	25
BMD 16	60	7	Del45-47	53

*Table displays age at follow-up, age at symptom onset, gene mutation, and disease duration in years*.

### Quantitative Muscle Strength Measure

Maximal voluntary isometric contraction of the trunk was acquired using Biodex (Biodex System 4 Pro, Biodex Medical Systems, Shirley, NY) with a Dual position back Extension/Flexion attachment (model number 830-450). The anterior iliac spine was aligned with the attachment's fixed axis of rotation, and back support was set at 100 degrees of hip angle. To minimize the influence of muscles from other parts of the body, chest and thighs were immobilized with Velcro straps, and the participants were asked to cross their arms in front of their chest during the test. Each participant was instructed to perform a maximal isometric trunk extension and trunk flexion. One contraction lasted 5 s. To ensure maximal contraction, two submaximal test trials were performed to familiarize the participants with the testing protocol for each position followed by three trials of maximal contractions (interchangeably between extension and flexion) with 30 s of rest in between each contraction. Standardized verbal encouragement was provided to each participant during testing.

### MRI Data Acquisition and Processing

**Figure 1:**
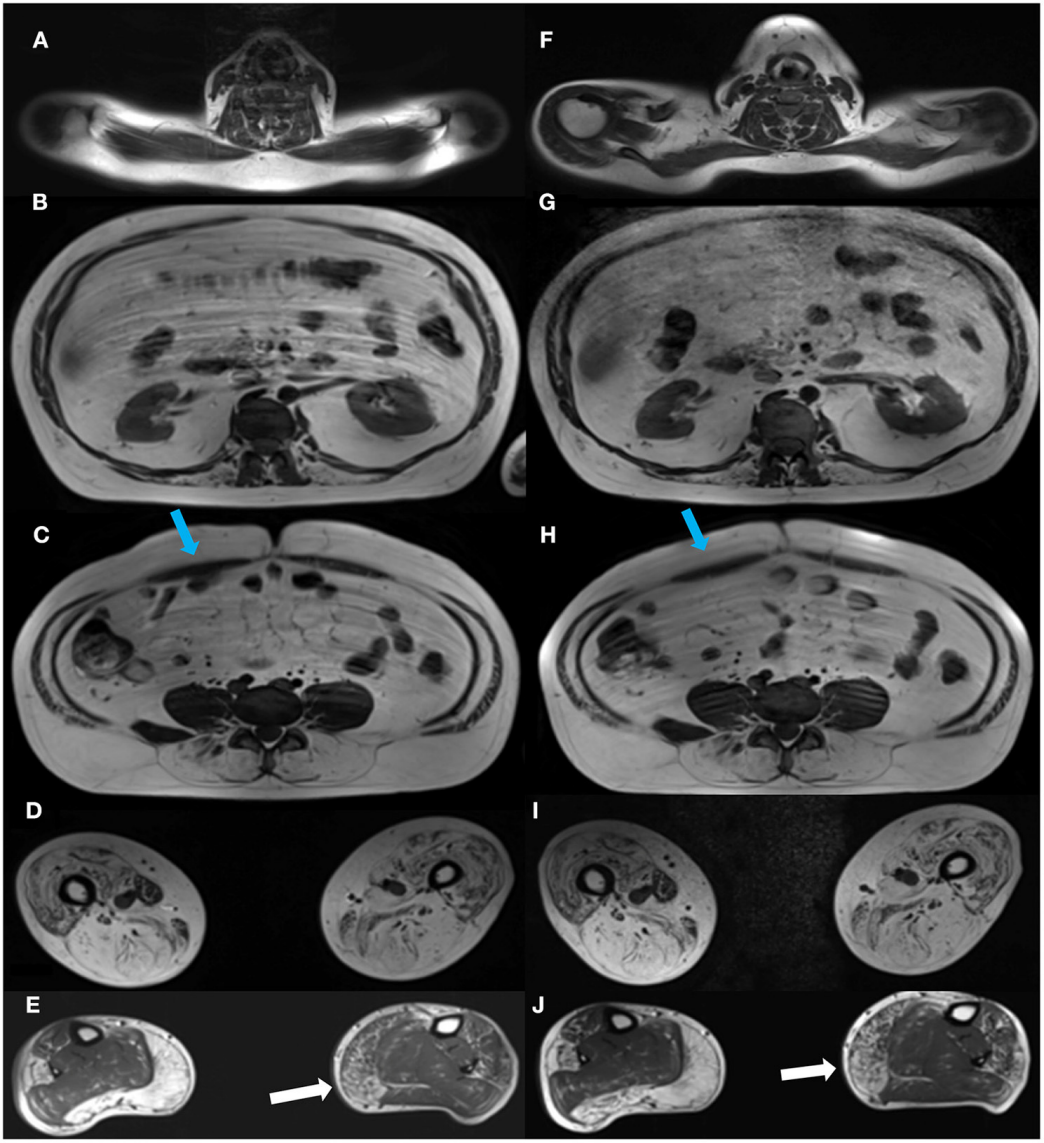
Cross-sectional MR images at baseline and at follow-up. Images of the cross-sectional slices at baseline (left column, **A–E**) and at follow-up (right column, **F–J**). Images are shown at spinal levels C6 **(A,F)**, T12 **(B,G)**, and L4/L5 **(C,H)**, thighs **(D,I)**, and lower legs **(E,J)**. A visual inspection of the images shows some progression of the right medial head of gastrocnemius from baseline, **(E)** to follow-up **(J)**, while changes in other muscles from baseline are indiscernible. Blue arrows show the abdominal muscles **(C,H)**.

Images were acquired on a Siemens 3.0 Tesla Magnetom Verio scanner (Erlangen, Germany) at the Department of Radiology at the National University Hospital, Rigshospitalet in Copenhagen, Denmark. Each participant was examined in head-first supine position. The MRI protocol composed of a three-plane localizer sequence followed by a T1-weighted sequence (field of view (FOV), 400–450 mm; slice thickness, 6.0 mm; distance factor, 20%; repetition time (TR)/echo time (TE), 19/650 ms), and a 2-point Dixon sequence (FOV, 400–450 mm; slice thickness, 3.5 mm; distance factor, 0%; TE/TR, 2.45 and 3.675/5.59 ms). Two body matrix coils and a peripheral leg coil were used for signal detection. Total scan time was approximately 40 min. Five cross-sectional slices were chosen for the investigation of disease progression (Figure 1). Dixon sequences were used to quantify fat fraction (FF) by defining a region of interest (ROI). Twenty-two ROIs were mapped bilaterally using Horos software v. 3.3.6 and consisted of the following muscles: Erector spinae (ES) was mapped at spinal level C6, Th12, and L4/L5 and multifidus (M) at spinal levels T12 and L4/L5. Abdominal muscles were not mapped but instead inspected visually due to motion artifact from respiration. Iliopsoas (P) was mapped at spinal level L4/L5, and muscles of the thigh were mapped at mid-thigh, corresponding to 50% of the length of femur: Rectus femoris (RF), vastus lateralis (VL), vastus medialis (VM), vastus intermedius (VI), Sartorius (SA), gracilis (GR), the adductor muscles (AM) (adductor magnus and adductor longus), and hamstring muscles: semimembranosus (SM), semitendinosus (ST), and biceps femoris (BF). At the widest section of the lower leg, corresponding to about 1/3 of tibia from the knee down, the following muscles were mapped: Tibialis anterior (TA), peroneals (PER), tibialis posterior (TP), soleus (SOL), gastrocnemius lateralis (GL), and gastrocnemius medialis (GM).

The cross-sectional area was determined from the mapping followed by quantitative fat fraction estimation. Mean FF % was expressed as percentage fat (0–100%) = signal fat/(signal water + fat). Bilateral mean FF % was used in the analysis. Baseline visit, follow-up visit, including mapping of ROIs was performed by the same examiner.

We analyzed our MRI data in two parts: First analysis consisted of all participants (*N* = 16). Based on the total mean muscle FF % per participant, we divided the participants in two groups that were analyzed separately: One group consisted of lesser-affected participants (*N* = 5, FF % < 35%) and the other of more severely affected participants (*n* = 11, FF % > 35%). In addition, based on direct observation, the participants with FF % < 35% displayed the least difficulty with ambulation such as rising from chair and walking, while participants with FF % > 35% displayed much greater ambulation difficulty.

### Statistical Analysis

Statistical analysis was performed using SPSS v22. All values are mean ± SD, unless otherwise stated. The Mann–Whitney test was used to test the null hypothesis of no difference in FF% from baseline to follow-up and trunk strength from baseline to follow-up. The level of significance was set at *p* ≤ 0.05.

## Results

The 16 participants with genetically verified Becker muscular dystrophy were 37.1 ± 11.9 years old and had a BMI of 27.5 ± 6.3 at follow-up. The mean follow-up interval was 398.4 ± 32.1 days (range of 365–478 days).

### Trunk Muscle Strength From Baseline to Follow-Up

**Figure 2:**
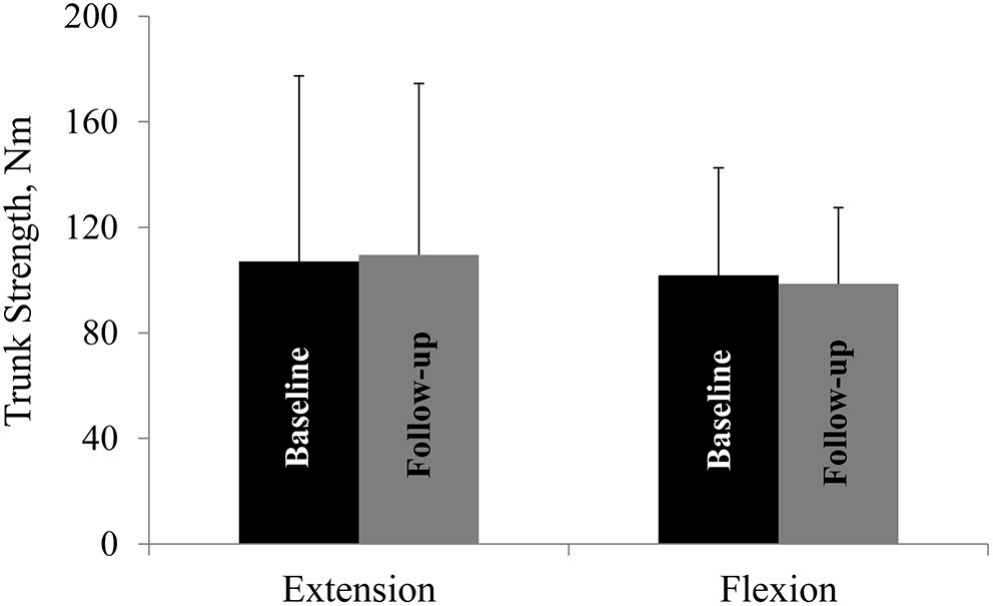
Trunk muscle strength at baseline and at follow-up. Trunk extension and flexion strength at baseline and at follow-up. Nm, Newton-meter. All values are mean ± SD.

Mean trunk muscle strength did not differ from baseline to follow-up in the whole cohort (Figure 2) or the two subgroups.

### Fat Fraction From Baseline to Follow-Up

**Figure 3:**
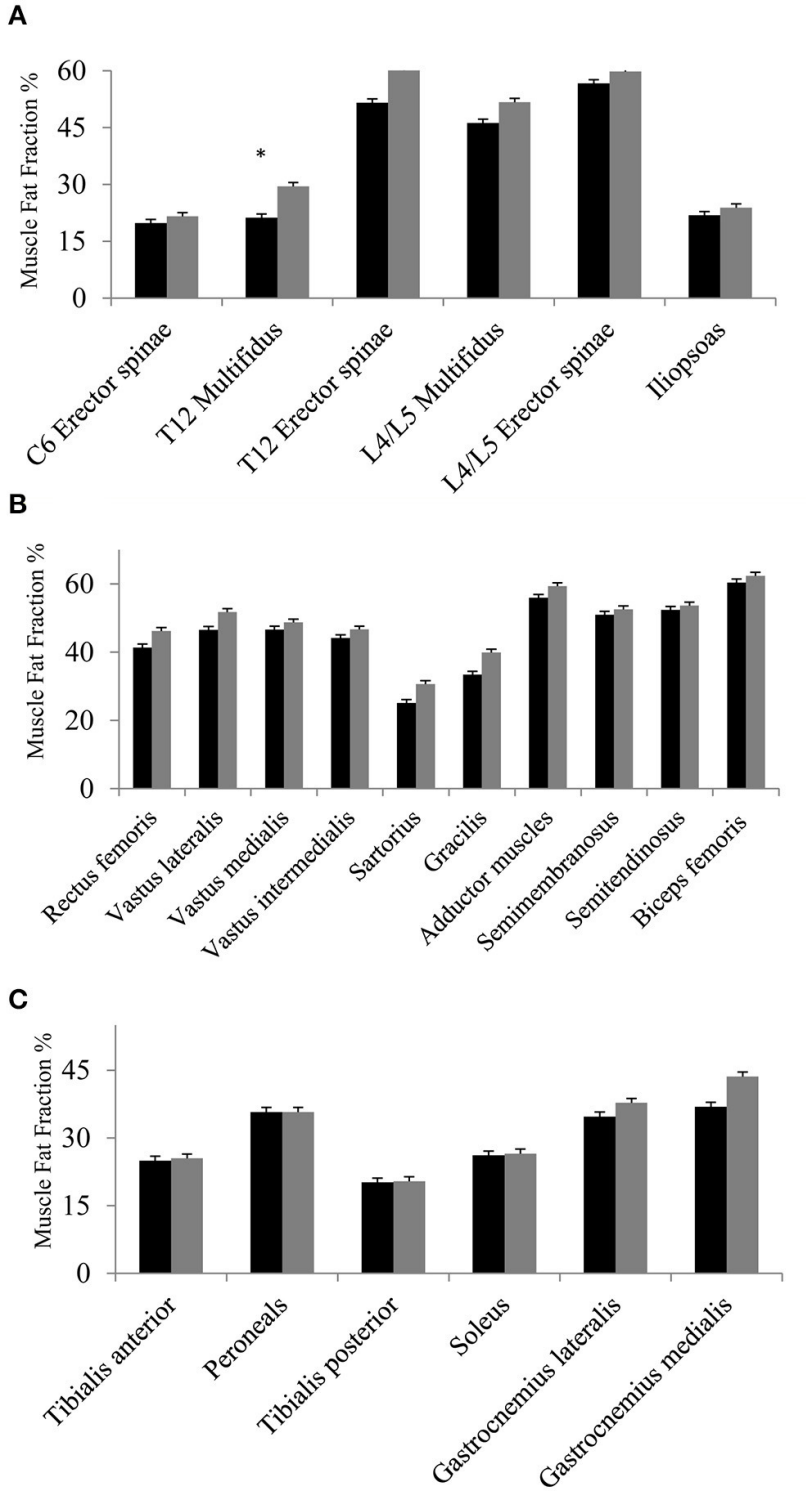
**(A–C)** Fat fraction % at baseline and at follow-up. Fat fraction % of muscles of the back and iliopsoas **(A)**, muscles of the thighs **(B)**, and muscles of lower legs **(C)** at baseline (black bars) and at follow-up (gray bars). Missing value for spinal level C6 (*n* = 1, due to phase-shift artifact), and missing value for thighs and lower legs (*n* = 1, due to positioning difficulty in scanner). Asterisk * indicates a significant difference (*p* = 0.01). All values are mean ± SD.

The first analysis showed no significant change in muscle FF% of the measured muscles of the back, thighs, and lower legs from baseline to follow-up, except for multifidus at spinal level T12 (Figures 3A–C), but all muscles showed a nominal increase in fat fraction from baseline to follow-up (3A–C). Abdominal muscles did not show any progression in fat replacement visually inspected at follow-up (Figure 1). The subgroup analysis showed a significant increase in FF% in the less affected group at L4/L5 ES (*p* = 0.047), SA (*p* = 0.028), GR (*p* = 0.009), TA (*p* = 0.047), PER (*p* = 0.028), and GM (*p* = 0.009) while the severely affected group increased significantly only at T12 M (*p* = 0.028) and T12 ES (*p* = 0.011).

## Discussion

This is the first study to examine longitudinal changes in disease progression in BMD using qMRI of the paraspinal muscles, lower limb muscles, and trunk muscle strength assessed by dynamometry. We found no significant change in trunk extension and flexion strength from baseline to follow-up, and qMRI on group level only identified a significant increase in FF% in the multifidus at spinal level T12. However, when we separately analyzed the lesser severe and severely affected participants, the FF% increased significantly in the lower back, sartorius, gracilis, and some of the lower leg muscles in the lesser affected individuals, while the severely affected participants only increased in FF% in their paraspinal muscle at spinal level T12. Irrespective of the significant changes in muscle FF%, muscle strength did not differ in the two subgroups.

We quantified trunk extension and flexion strength with a Biodex dynamometer. A previous study has established that strength measure reliability is excellent for trunk extension and moderate for trunk flexion (7). The absence of any change in trunk strength at follow-up, suggests that qMRI, which showed numerical increase in muscle FF%, is a more sensitive marker of change than strength. This discrepancy between qMRI progression and maintained muscle strength is in line with findings in other muscular dystrophies (8, 9) and is likely explained by the fact that the paraspinal muscles work as a whole unit and that muscle strength of a subset of the paraspinal musculature cannot be measured clinically.

On group level, we did not find any significant difference in FF% from baseline to follow-up, except at spinal level T12 multifidus. In contrast, a significant difference in muscle FF% has been found in 1-year follow-up of other muscular dystrophies (8, 9). One study found a significant difference in all measured muscles of the back, hip flexors, knee extensors and flexors, and lower leg muscles at 1-year follow-up in patients with facioscapulohumeral muscular dystrophy (8), and Willis et al. found a significant increase in FF% in 9 of 14 investigated muscles, including the hamstrings, sartorius, gracilis, vastus lateralis, rectus femoris, and calf muscles in limb–girdle muscular dystrophy type R9 (9). This could suggest that the rate of disease progression in BMD is slower than these other muscular dystrophies and that a 1-year follow-up therefore may be too short to show change. However, our participants had a higher FF% at baseline and therefore there could have been a ceiling effect for change in FF%. In support of this notion, the lesser-affected group of BMD participants significantly increased their muscle FF% in 6 of 22 assessed muscles at follow-up, while the severely affected participants only increased in FF% in one. This suggests that disease progression in BMD may not be much different from FSHD and LGMDR9, when patients with the same disease severity are compared. It also highlights the importance of stratifying patients with BMD according to phenotype for future clinical trials. In addition, the small number of participants in our study may partly explain the lack of significant progression in FF% on a group level.

Another limitation to our study may be the use of 2-point Dixon imaging. Two-point Dixon has shown to be sensitive to phase-shift artifacts in comparison with 3-point Dixon (10); however, we only experienced this with one subject at spinal level C6 and those images were not included in the analysis.

A recent study reported that increases in T2 heterogeneity were observed in fat-replaced muscles in relation to increased disease activity (11). Therefore, it may be of interest to add T2 to MRI protocols in future studies to expand the understanding of disease activity.

In conclusion, we recommend that for future trials patients with BMD should be stratified according to phenotype because they evolve differently and that there is an emphasis on including mild to moderately affected patients, because capturing a change in disease progression in severely affected patients may not be as evident as the lesser affected patients.

## Data Availability Statement

The original contributions presented in the study are included in the article/supplementary materials, further inquiries can be directed to the corresponding author/s.

## Ethics Statement

The studies involving human participants were reviewed and approved by Danish National Committee on Health Research Ethics (approval number: H-16030358). The patients/participants provided their written informed consent to participate in this study.

## Author Contributions

AMS contributed with data collection, data analysis, and drafting of manuscript. KR contributed with study design, data collection, and drafting of manuscript. NW contributed with study design and drafting of manuscript. JV contributed with study design, data analysis, and drafting of manuscript. All authors contributed to the article and approved the submitted version.

## Conflict of Interest

The authors declare that the research was conducted in the absence of any commercial or financial relationships that could be construed as a potential conflict of interest. The reviewer GT declared a past co-authorship with one of the authors JV to the handling Editor.

## References

[1] Tasca G Iannaccone E Monforte M Masciullo M Bianco F Laschena F. Muscle MRI in Becker muscular dystrophy. Neuromuscul Disord NMD. (2012) 22 (Suppl. 2):S100–6. 10.1016/j.nmd.2012.05.015 22980760

[2] Faridian-Aragh N Wagner KR Leung DG Carrino JA. Magnetic resonance imaging phenotyping of Becker muscular dystrophy. Muscle Nerve. (2014) 50:962–7. 10.1002/mus.24246 24659522

[3] Bonati U Schmid M Hafner P Haas T Bieri O Gloor M. Longitudinal 2-point dixon muscle magnetic resonance imaging in becker muscular dystrophy. Muscle Nerve. (2015) 51:918–21. 10.1002/mus.24629 25736228

[4] Maggi L Moscatelli M Frangiamore R Mazzi F Verri M De Luca A. Quantitative muscle MRI protocol as possible biomarker in Becker muscular dystrophy. Clin Neuroradiol. (2020). 10.1007/s00062-019-00875-0. [Epub ahead of print]. 31974637

[5] Harbo T Brincks J Andersen H. Maximal isokinetic and isometric muscle strength of major muscle groups related to age, body mass, height, and sex in 178 healthy subjects. Eur J Appl Physiol. (2012) 112:267–75. 10.1007/s00421-011-1975-3 21537927

[6] Zouita S Ben Salah FZ Behm DG Chaouachi A Isokinetic trunk strength, validity, reliability, normative data and relation to physical performance and low back pain: a review of the literature. Int J Sports Phys Ther. (2020) 15:160–74. 10.26603/ijspt20200160 32089967 PMC7015027

[7] Rudolf K Sheikh A Knak K Witting N Vissing J EP.54Assessment of trunk muscle strength in patients with muscular dystrophies using stationary and hand-held dynamometry: a test-retest reliability study. Neuromuscul Disord. (2019) 29:S116–7. 10.1016/j.nmd.2019.06.286

[8] Andersen G Dahlqvist JR Vissing CR Heje K Thomsen C Vissing J. MRI as outcome measure in facioscapulohumeral muscular dystrophy: 1-year follow-up of 45 patients. J Neurol. (2017) 264:438–47. 10.1007/s00415-016-8361-3 28000006

[9] Willis TA Hollingsworth KG Coombs A Sveen M-L Andersen S Stojkovic T. Quantitative muscle MRI as an assessment tool for monitoring disease progression in LGMD2I: a multicentre longitudinal study. PLoS ONE. (2013) 8:e70993. 10.1371/journal.pone.0070993 23967145 PMC3743890

[10] Coombs BD Szumowski J Coshow W. Two-point Dixon technique for water-fat signal decomposition withB0 inhomogeneity correction. Magn Reson Med. (1997) 38:884–9. 10.1002/mrm.1910380606 9402188

[11] Hooijmans MT Froeling M Koeks Z Verschuuren JJGM Webb A Niks EH. Multi-parametric MR in Becker muscular dystrophy patients. NMR Biomed. (2020) 33:e4385. 10.1002/nbm.4385 32754921 PMC7687231

